# Mouse Model of Devil Facial Tumour Disease Establishes That an Effective Immune Response Can be Generated Against the Cancer Cells

**DOI:** 10.3389/fimmu.2014.00251

**Published:** 2014-05-27

**Authors:** Terry L. Pinfold, Gabriella K. Brown, Silvana S. Bettiol, Gregory M. Woods

**Affiliations:** ^1^Menzies Research Institute Tasmania, University of Tasmania, Hobart, TAS, Australia; ^2^School of Medicine, University of Tasmania, Hobart, TAS, Australia

**Keywords:** Tasmanian devil, transmissible cancer, mouse model, immunogenicity, Devil Facial Tumour Disease

## Abstract

The largest carnivorous marsupial in Australia, the Tasmanian devil (*Sarcophilus harrisii*) is facing extinction in the wild due to a transmissible cancer known as Devil Facial Tumour Disease (DFTD). DFTD is a clonal cell line transmitted from host to host with 100% mortality and no known immunity. While it was first considered that low genetic diversity of the population of devils enabled the allograft transmission of DFTD recent evidence reveals that genetically diverse animals succumb to the disease. The lack of an immune response against the DFTD tumor cells may be due to a lack of immunogenicity of the tumor cells. This could facilitate transmission between devils. To test immunogenicity, mice were injected with viable DFTD cells and anti-DFTD immune responses analyzed. A range of antibody isotypes against DFTD cells was detected, indicating that as DFTD cells can induce an immune response they are immunogenic. This was supported by cytokine production, when splenocytes from mice injected with DFTD cells were cultured *in vitro* with DFTD cells and the supernatant analyzed. There was a significant production of IFN-γ and TNF-α following the first injection with DFTD cells and a significant production of IL-6 and IL-10 following the second injection. Splenocytes from naïve or immunized mice killed DFTD cells in *in vitro* cytotoxicity assays. Thus, they are also targets for immunological destruction. We conclude that as an immune response can be generated against DFTD cells they would be suitable targets for a vaccine.

## Introduction

The Tasmanian devil (*Sarcophilus harrisii*) faces possible extinction in the wild due to a transmissible cancer known as Devil Facial Tumour Disease (DFTD). Genetic and chromosomal research has provided convincing evidence that the malignant neoplasm originated in an individual female Tasmanian devil (1). While it was first considered that low genetic diversity of the population of devils, from which the DFTD founder was derived, enabled the establishment of DFTD throughout subsequent devil populations (2, 3), recent evidence reveals that genetically diverse animals are prone to the disease (4).

The unique and conserved chromosomal rearrangements of DFTD cells compared to the host’s negate the possibility of transmission from a viral or bacterial agent, pollutants or toxins in the environment. DFTD is described, alongside the canine transmissible venereal tumor (CTVT), as a clonal cell line immortalized as a parasitic infectious allograft (5). Not only did the DFTD cancer cells evade the original host’s immune system but the immune systems of subsequent devils. The cancer cells are transmitted through facial biting of successive hosts (1, 6, 7).

A lack of immunogenicity is one possible mechanism for DFTD cells to evade the immune system. A failure to present tumor antigens to the immune system would facilitate transmission between devils. Siddle et al. (8) revealed that DFTD cells do not express cell surface MHC molecules *in vitro* or *in vivo*. The genes essential to the antigen-processing pathway, such as β_2_-microglobulin and transporters associated with antigen processing are down-regulated. The loss of gene expression is not due to structural mutations, but to regulatory changes including epigenetic deacetylation of histones (8). By down-regulating MHC, the tumor cells remain invisible to the devils’ immune system. But even in the absence of MHC expression, there should be enough protein differences to induce an immune response following allogenic transfer.

In order to develop an effective vaccine or immunotherapy, it is important to understand the mechanisms that shield this tumor from immunosurveillance. Ideally, an immunological study of an allograft tumor in the host species is necessary, but in the case of the Tasmanian devil conducting large scale immunological experiments are not possible due to the endangered species status. Therefore, since it is widely accepted that mouse models provide valuable insights into the study of human cancers our investigation exploits a mouse model to study DFTD. The particular advantage of a mouse model is the readily available antibodies to detect mouse immune system responses while there is a paucity of equivalent antibodies currently available for the Tasmanian devil immune system.

To determine if DFTD cells are immunogenic and therefore potential targets for immunotherapy, we used a mouse model developed in our laboratory to examine DFTD. We have previously shown that the tumors successfully implant in immunocompromised NOD/SCID mice but do not implant in immunocompetent BALB/c and C57BL/6 mice. The aim was to determine if this rejection by immunocompetent mice was an active immunological response and not due to other factors such as preformed antibodies commonly associated with xenogeneic graft rejection. This was performed by examining specific antibody, cytokine, and cell mediated cytotoxicity responses to the DFTD xenograft.

In addition, this xenograft model was used to investigate the possibility that if DFTD cells are immunogenic they may polarize the murine immune system toward a T_H_2 response. This is a mechanism exploited by tumor cells to subvert anti-tumor immune responses toward ineffective humoral responses (9).

## Materials and Methods

### Mice

All mice were obtained from the University of Tasmania Central Animal House and housed in a pathogen free environment and provided with food and water *ad libitum*. The two strains used were C57BL/6 and BALB/c. The mice were at least 5 weeks old at commencement of experimental treatments.

All animal experiments were performed with approval of the Animal Ethics Committee of University of Tasmania (UTAS), approval numbers A0010231 and A0010888.

### Cell lines and culture media

Devil Facial Tumour Disease cell line, C5065, was obtained from stocks stored in liquid nitrogen at the UTAS. The cell line was established from primary tumor biopsy samples taken under the approval of the Animal Ethics Committee of Tasmania’s Park and Wildlife Services (permit numbers 33/2004–5 and 32/2005–6) and provided by A.-M. Pearse and K. Swift, Tasmanian Department of Primary Industries, Parks, Wildlife and Environment (DPIPWE). Cells were grown in RPMI-10FCS, which consisted of RPMI-1640 medium (GIBCO, New York, USA) supplemented with 10% fetal calf serum (FCS) (Bovogen Biological, VIC, Australia), 5 mM l-glutamine (Sigma-Aldrich, St Louis, MO, USA), and 40 mg/ml of gentamicin (Pfizer, Bentley Australia). The C5065 cell line culture was maintained in a fully humidified 5% CO_2_ incubator at 35°C. Assays required co-culturing of mouse splenocytes with C5065 were maintained at 37°C.

### Indirect immunofluorescence assay for detection of antibodies by flow cytometry

Blood was collected from mice post mortem via cardiac puncture or via mandible bleed with living mice, allowed to clot and the serum was separated by centrifuging. Serum was stored at −20 or −80°C until required. One microliter of each serum sample was incubated with 10^5^ DFTD cells in 50 μl of staining buffer [phosphate-buffered saline (PBS) with 1% (w/v) bovine serum albumin (BSA) (Roche Diagnostics, Germany) and 0.01% NaN_3_], on ice for 30 min. Following two washes with PBS, the cells were incubated with 2 μg of the relevant anti-mouse immunoglobulin Alexa-fluor conjugated antibody (Life Technologies, USA, catalog numbers IgG A-31553, IgG_1_ A-21121, IgG_2a_ A-21136, IgG_2b_ A-21146, IgG_3_ A-21151, IgM A-21042) in 100 μl of staining buffer on ice for 30 min. Negative controls included DFTD cells with either no serum or pooled naïve serum. Following three washes in PBS, the samples were resuspended in 100 μl of PBS for prompt reading on the flow cytometer (BD Canto II, Becton Dickinson, NJ, USA).

The forward scatter, side scatter, thresholds, and PMT voltages were optimized to place the negative mean fluorescence intensity (MFI) for the negative controls at approximately the second decade of the log scale. FCS 4 Express Flow Cytometry software (De nova Software, USA) was used to perform histogram overlays to compare indirect immunofluorescence levels of samples with naïve serum control. The geometric mean of the MFI for immune serum was divided by the geometric mean of the MFI for naïve serum control to give the relative antibody levels.

### Immunizations

Devil Facial Tumour Disease cells in log growth phase were harvested from culture and viability w*as* ascertained by trypan blue exclusion. Cell viability ranged from 50 to 80%. Cells were washed, resuspended in PBS, and 2 × 10^6^ viable cells were injected via the subcutaneous (SC) route or intraperitoneal (IP) route into C57BL/6 and BALB/c mice. With multiple sites SC injections, the cells were divided between multiple sites as stated. No adjuvants were used.

### Mouse mononuclear cells for *in vitro* cytokine and cytotoxicity assays

Spleens were removed from naïve or IP immunized mice. Lymph nodes were obtained from SC immunized mice. Mononuclear cells (MNC) were obtained by pressing spleens or lymph nodes through 40 μm cell strainers (BD Falcon, USA) and then separating them by density-gradient centrifugation on Histopaque-1083 gradient (Sigma-Aldrich, USA) following the manufacturer’s protocol. The MNC were washed twice with PBS and resuspended in RPMI-10FCS for use in *in vitro* cytotoxicity and cytokine assays.

### *In vitro* cytokine cultures from splenocyte supernatants

Mononuclear cells were prepared as described above and resuspended in RPMI-10FCS at 10^7^ cells/ml. DFTD cells were harvested and resuspended at 10^5^ cells/ml in RPMI-10FCS. One hundred microliters each of DFTD and lymphocyte suspensions were combined in V-bottomed 96 well plates (Greiner Bio-one, Frickenhausen, Germany). Control wells were also prepared in the same plate by combining 100 μl RPMI with 100 μl of either DFTD or MNC as indicated. The plates were covered by lids and incubated for 72 h at 37°C with 5% CO_2_. The plates were centrifuged to pellet the cells and the supernatant collected and stored at −20°C until required. Supernatant samples were assayed individually for cytokine activity using BD Biosciences CBA T_H_1, T_H_2, T_H_17 micro-bead array kit and FACS array software as per manufacturer’s instruction (BD Biosciences, Cat # 560485). In brief, 50 μl of each supernatant sample and cytokine standards were incubated with mixed capture beads and PE detection reagent for 3 h, washed, resuspended, and run on the BD Canto II, flow cytometer. Cytokine data were analyzed using FCAP Array software (BD Bioscience, San Jose, CA, USA).

### Cytotoxicity assay using immunofluorescence double staining protocol

DFTD (10^7^; target cells) were labeled with 5(6)-Carboxyfluorescein diacetate *N-succinimidyl* ester (CFSE) by incubating with 2 μl of 5 mM CFSE for 30 min at 23°C on shaker plate protected from light. Cells were washed twice and resuspended at 10^5^ cells/ml in RPMI-10FCS.

Mononuclear cells were resuspended in RPMI-10FCS at 10^7^ cells/ml. A V-bottomed 96 well plate (Greiner Bio-one, Frickenhausen, Germany) was prepared with 100 μl serial dilutions of the MNC to provide effector ratios of 100:1, 50:1, 25:1, 12:1, 6:1, and 3:1 with 10^4^ target cells.

The cytotoxicity assays were performed using four replicate samples and incubating for 18 h at 37°C in a fully humidified 5% CO_2_ incubator. The plates were centrifuged for 5 min at 500 *g*, supernatant removed, pellet resuspended in 100 μl PBS + 1 μl propidium iodide (PI), and read on a BD Canto II flow cytometer with the High Throughput Sampler unit directly recovering samples from the wells.

Flow cytometry data analysis was performed using Flowing Software (Turku Centre for Biotechnology, Finland), or FCS 4 Express Flow Cytometry (De Nova Software, USA), with further analysis and graphs produced in Microsoft Excel 2007 and GraphPad Prism 5.

Cells positive for CFSE and PI were identified as dead target cells whereas cells positive for CFSE, but negative for PI, were identified as viable target cells. Cytotoxicity per well was calculated as dead target cells divided by the sum of dead and viable target cells and expressed as a percentage. The average cytotoxicity for each effector target ratio was calculated and the standard error of the mean (SEM) values was used for the error bars.

### Statistical analyses

Quantitative data comparing two groups are expressed as the mean ± SEM and *P*-values calculated using Student’s unpaired two tailed *t*-test. Quantitative data comparing more than two groups are expressed as the mean with the probability calculated by one-way ANOVA followed by Dunnett’s test.

## Results

### Naïve BALB/c and C57/BL6 mice have barely detectable levels of preformed antibodies but following immunizaton have high levels of specific antibodies

**Figure 1:**
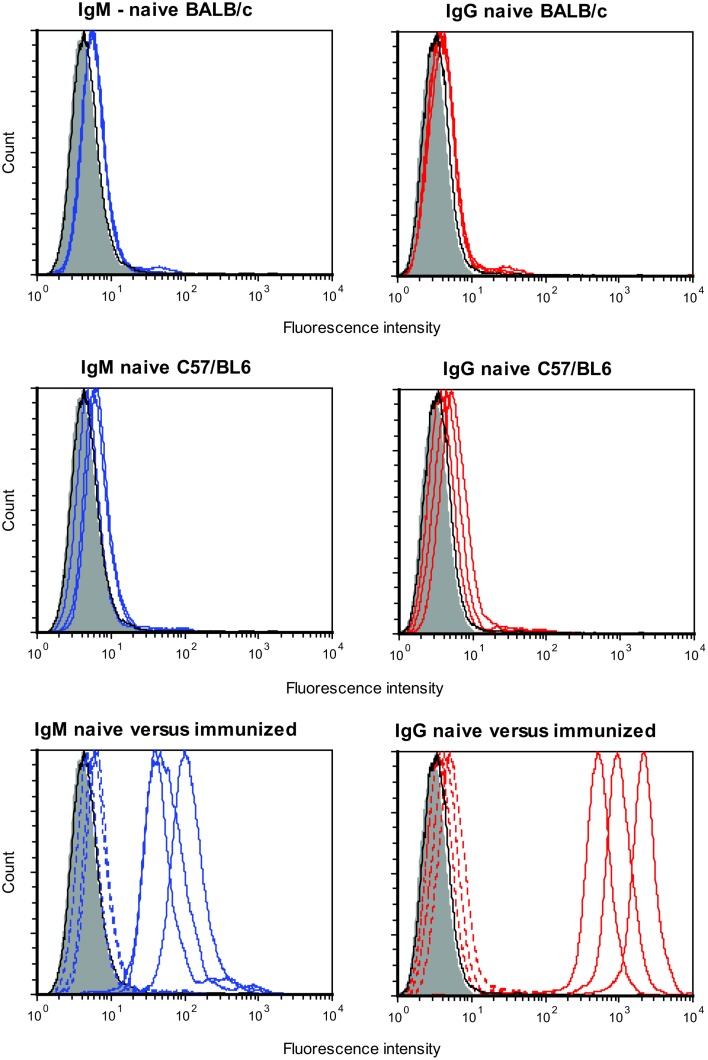
**Serum from three naïve BALB/c mice (upper panel) and three naïve C57/BL6 mice (middle panel) were analyzed for preformed IgM (left panel) and preformed IgG (right panel) antibodies that bound DFTD cells**. Three BALB/c mice were then immunized intraperitoneally with DFTD cells and specific IgM and IgG antibodies were analyzed (lower panel). Shaded areas represent no primary antibody, solid black lines represents isotype (IgM or IgG) control, blue lines represent IgM anti-DFTD antibodies, red lines represent IgG anti-DFTD; lower panel, dotted lines represent serum from naïve mice, solid lines represent serum from mice immunized intraperitoneally with DFTD.

There was no evidence for substantial levels of preformed antibodies as serum from naïve mice showed minimal binding to DFTD cells. This was the case for both IgG and IgM. As shown in Figure 1, flow cytometry profiles of serum from three representative naïve BALB/c and C57/BL6 mice show barely detectable levels of preformed antibodies. When immunized intraperitoneally, both strains of mice had considerable increases in levels of specific antibodies to DFTD cells. As future experiments were aimed at evaluating specific immunity following DFTD cell immunization, the results were summarized as histograms rather than displaying multiple flow cytometry plots. The geometric mean of the MFI of the serum from immunized mice was divided by the geometric mean of the MFI of the serum from naïve mice.

### Subcutaneous injections of BALB/c mice with DFTD cells induces antibody production

**Figure 2:**
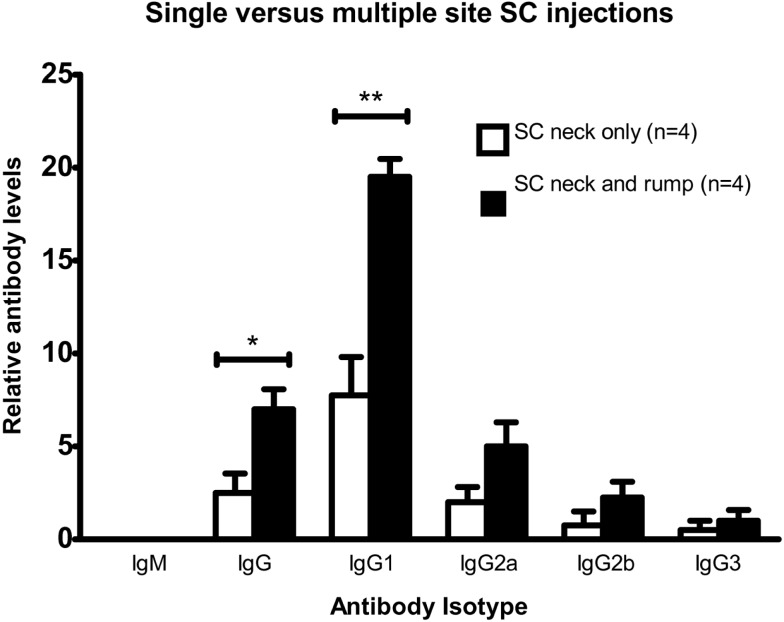
**Comparison of antibody responses of BALB/c mice injected twice subcutaneously with 10^6^ DFTD cells into a single site, or divided equally between two sites on days 0 and 16, with serum collected day 25**. Mean fluorescence intensity ratio of samples compared to pooled naïve mouse serum was used as a relative measure of antibody production. IgM was not detected in any sample (Data are expressed as mean ± SEM; probability calculated by an unpaired Student’s *t*-test *P < 0.05, **P < 0.01).

BALB/c mice were twice injected subcutaneously with viable DFTD cells, which were obtained from cell culture. The objective was to determine if living DFTD cells could elicit an antibody response. Flow cytometry was used to analyze serum for IgG and IgM antibodies specific to DFTD surface antigens. All the IgG isotypes tested for were detected, but IgM was not detected (Figure 2), thus indicating that DFTD cells can induce an antibody response. The responses to single site SC injections were compared with multiple site SC injections to determine if multiple site injections with the same number of total cells produced a greater response. Multiple site SC injections produced greater total IgG (*P* < 0.05; unpaired Student’s *t*-test) and IgG1 (*P* < 0.01; unpaired Student’s *t*-test) responses than injecting the same number of DFTD cells into a single site (Figure 2).

### Intraperitoneal injections of C57BL/6 mice with DFTD cells induce greater antibody responses than SC injections

**Figure 3:**
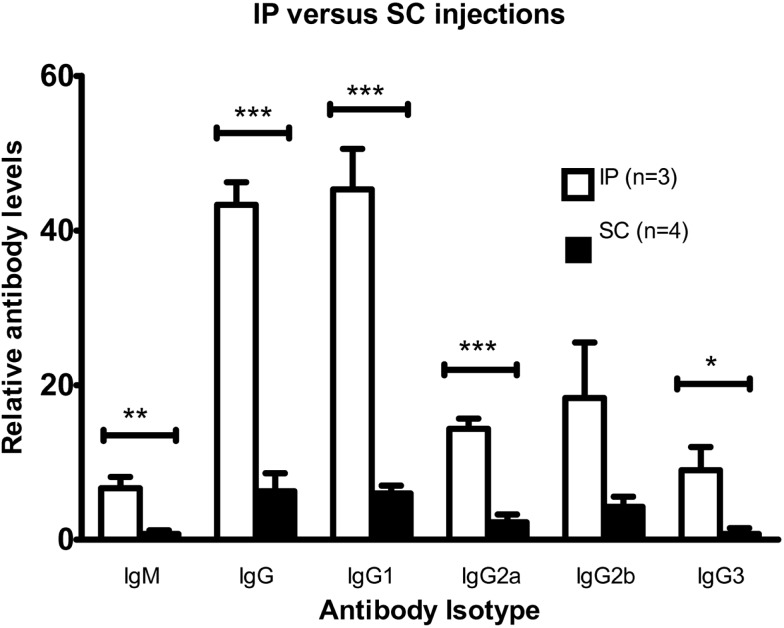
**Comparison of antibody responses for intraperitoneal and multiple sites subcutaneous injections with DFTD cells**. Mean fluorescence intensity ratio of samples compared to pooled naïve mouse serum was used as a relative measure of antibody expression. C57BL/6 mice were injected with 10^6^ DFTD cells on day 0 and 16 with serum collected on day 25. The SC cohort received SC injections divided between the neck and rump region to target multiple draining lymph nodes (Data are expressed as mean ± SEM; probability calculated by an unpaired Student’s *t*-test, *P < 0.05, **P < 0.01, and ***P < 0.001).

C57BL/6 mice were intraperitoneally or subcutaneously injected twice with viable DFTD cells. The objective was to determine, which route produced a better antibody response. Results in Figure 3 show that the IP route produced both IgM and IgG antibodies. The responses following the IP injections were stronger than the SC route for IgM (*P* < 0.01; unpaired Student’s *t*-test), total IgG, IgG1, IgG2a (*P* < 0.001; unpaired Student’s *t*-tests), and IgG3 (*P* < 0.05; unpaired Student’s *t*-test).

### A single intraperitoneal injection of C57BL/6 mice with DFTD cells induces both IgM and IgG antibody responses within 7 days

**Figure 4:**
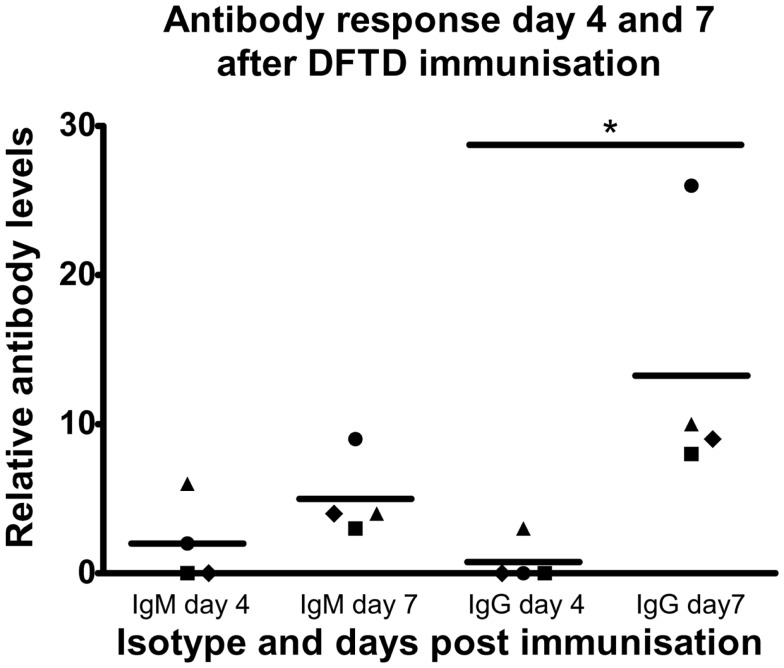
**C57BL/6 mice were injected IP with 10^6^ DFTD cells and serum was collected 4 and 7 days post injection**. All samples were analyzed in the same flow cytometry experiment to permit direct comparison of mouse anti-DFTD IgG and IgM in the serum. Mean fluorescence intensity ratio of samples compared to pooled naïve mouse serum was used as a relative measure of antibody expression. There was no significant difference in the mean of IgM expression for all four mice (represented by different plot symbols) compared on days 4 and 7 post injection. There was a significant difference of IgG expression between days 4 and 7 (*P* < 0.05; probability calculated by a paired Student’s*t*-test).

Having established that immunization with DFTD cells induces strong IgG responses following two injections, we next determined if IgG and IgM responses could be detected after a single injection. Results in Figure 4 show 4 days was insufficient to generate optimum levels of antibody in the serum with IgM detected in only two of the four mice and IgG detected in only one of these two mice. By 7 days all mice produced both IgM and IgG antibodies with the predominant isotype being IgG (*P* < 0.05; Student’s unpaired *t*-test) (Figure 4).

### Secondary antibody responses demonstrate enhanced memory responses for IgG but not IgM

**Figure 5:**
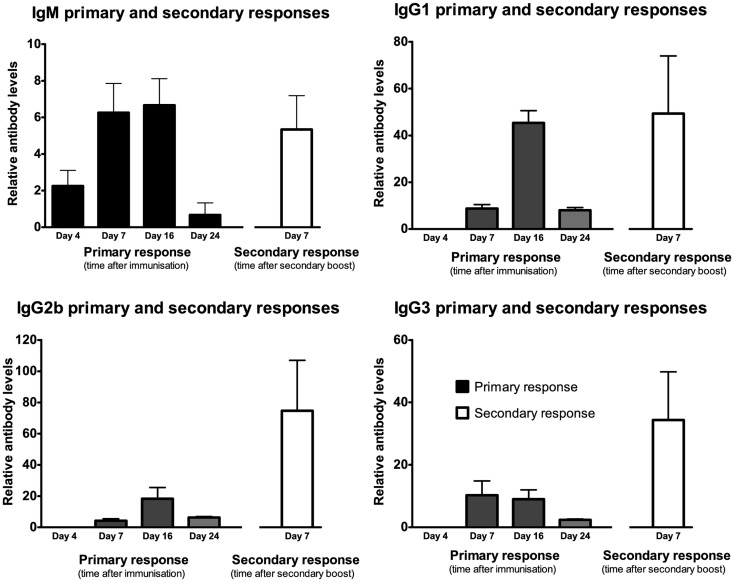
**Following a single IP injection serum samples were collected on day 4, 7, 16, and 24 (results shown in black)**. Secondary immunization occurred on day 95 and serum was collected 7 days later (results shown in white). Mean fluorescence intensity ratio of samples compared to pooled naïve mouse serum was used as a relative measure of antibody expression. Data expressed as mean of three mice ± SEM except day 4 and 7 primary response (*n* = 4).

Responses to secondary immunizations following 95 days showed enhanced responses in IgG1, IgG2b, and IgG3 antibodies but not IgM (Figures 4 and 5). The enhanced response is consistent with a primed immune system memory response because it was evident 95 days after the primary immunization.

### Intraperitoneal injections of DFTD cells do not induce greater cytotoxicity in splenocytes than those obtained from naïve mice

**Figure 6:**
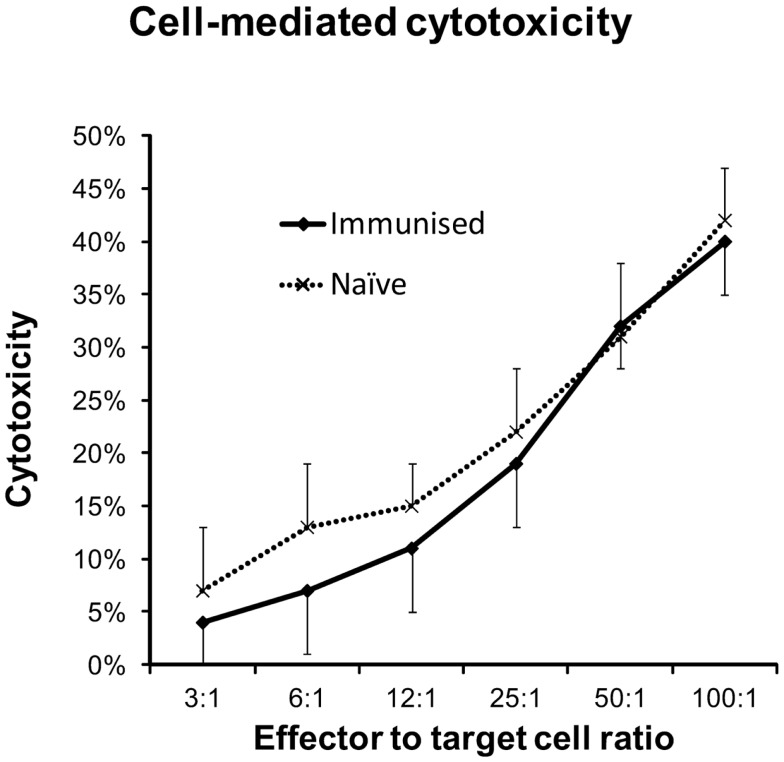
**Splenocytes from naïve and immunized C57BL/6 mice were evaluated for cytotoxic responses against DFTD cells**. Immunized mice were given IP injections on days 0 and 14 and splenocytes harvested on day 21. DFTD target cells were labeled with CFSE and dead cells identified with PI staining. Flow cytometry was used to calculate cell mediated cytotoxicity as a percentage of CFSE labeled DFTD target cells. Splenocytes from naïve (*n* = 4) or immunized (*n* = 5) mice and DFTD target cells were incubated for 18 h *in vitro* at 37°C with 5% CO_2_. Results represent the mean and standard deviation of the combined cytotoxicities of all mice with four technical replicates for each mouse.

As IP injections of DFTD cells produced the best antibody response, we used this route to determine if an enhanced cytotoxic response could be induced against DFTD cells. Splenocytes from naïve C57BL/6 mice demonstrated dose response cytotoxicity against DFTD cells following 18 h incubation (Figure 6). This was not improved following two IP injections with DFTD cells, indicating that unprimed cells of the immune system mediated the cytotoxicity.

### Immunization with DFTD cells does not bias antibody or cytokine responses toward a T_H_2 profile

Since tumor cells can weaken anti-tumor immune responses by skewing them toward less effective T_H_2 responses, we examined the antibody and cytokine profiles for such a bias. Two strains of mice were compared because of their reportedly opposing T_H_1 (C57BL/6) or T_H_2 (BALB/c) dominated immune responses (10, 11).

**Figure 7:**
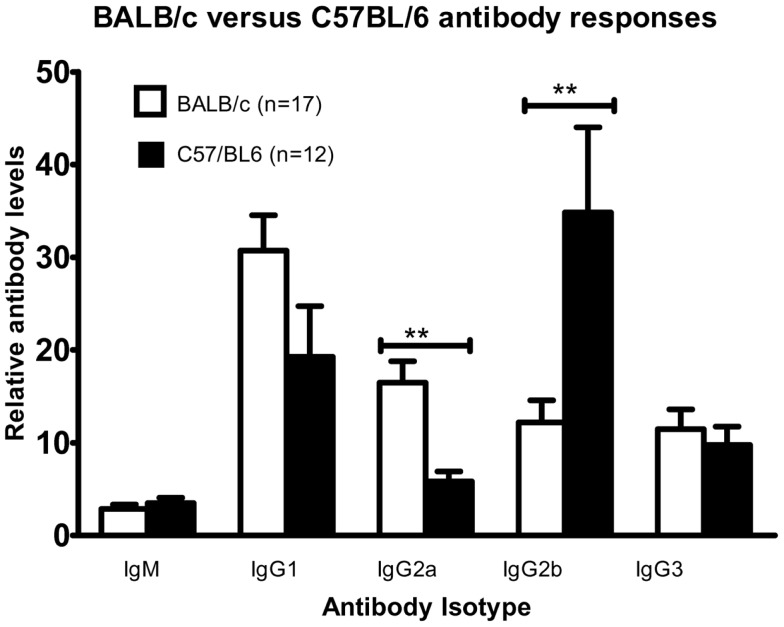
**Cohorts of BALB/c and C57BL/6 mice were injected with 10^6^ DFTD cells by IP injection on Day 0, with a second injection between day 14 and 16; serum was collected 7–8 days later**. While anti-DFTD antibody expression varied between individuals there was a consistent trend that BALB/c mice were skewed toward higher levels of IgG2a and lower levels of IgG2b compared to C57BL/6 mice (Data are expressed as mean ± SEM, probability calculated by unpaired Student’s *t*-test, **P < 0.01).

Mice were injected intraperitoneally twice with DFTD cells and serum was collected 7–8 days following the second immunization. Levels of IgM, the T_H_2 antibody, IgG1, and the T_H_1 antibody IgG3 were not significantly different between the two strains (Figure 7). Of the other T_H_1 antibodies, BALB/c mice expressed higher levels of IgG2a and lower levels of IgG2b compared to C57BL/6 (*P* < 0.05; unpaired Student’s *t*-test).

As up-regulation of certain cytokines in the tumor microenvironment can enhance or suppress tumor rejection, we next assessed the cytokine production of splenocytes from naïve mice, mice given a single IP injection of DFTD and mice given two IP injections of DFTD cells. Splenocytes were obtained from the mice and incubated *in vitro* for 72 h with DFTD cells and the supernatant analyzed for cytokines. Cytokine levels expressed into the growth media were then measured.

**Figure 8:**
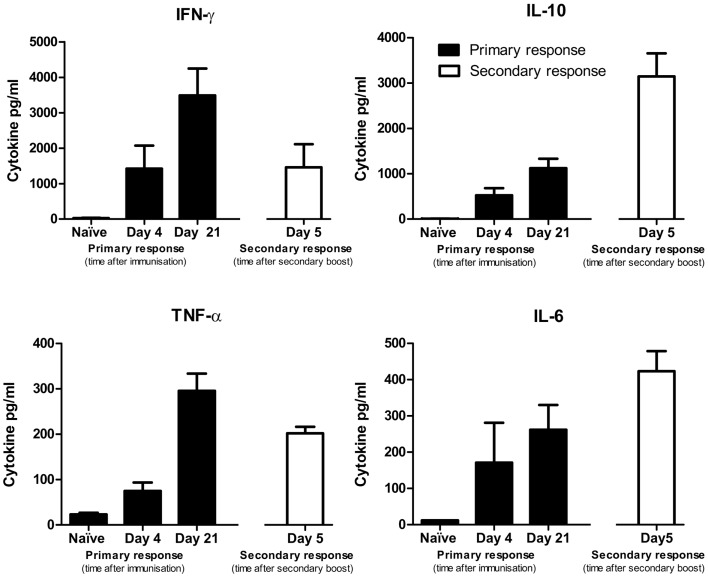
**Cytokine production by splenocytes from naïve mice or DFTD immunized mice, cultured *in vitro* with DFTD cells for 72 h**. Primary responses refer to mice given a single injection of 2 × 10^6^ DFTD cells and day refers to time post immunization. Secondary responses refer to mice immunized with 2 × 10^6^ DFTD cells, rested 57 days, given a second immunization with 2 × 10^6^ DFTD cells and splenocytes collected 5 days later. Data expressed as mean of five mice ± SEM except naïve responses (*n* = 3).

Splenocytes from non-immunized mice did not produce detectable cytokines. Evidence for IFN-γ, TNF, IL-6, and IL-10 production was apparent for splenocytes obtained at 4 and 21 days following a single immunization (Figure 8). These same cytokines were also detected when splenocytes were obtained 5 days after a secondary DFTD immunization.

Of the T_H_1 cytokines, peak levels of IFN-γ and TNF-α were detected from splenocytes obtained at day 21 after a single immunization. Peak levels of the T_H_2 cytokine IL-10 were detected following a secondary immunization. The pro-inflammatory cytokine IL-6 followed a similar pattern to IL-10 with peak values occurring after a secondary immunization. IL-4 and IL-12 were not produced at detectable levels and are therefore not shown. The antibody and cytokine profiles did not suggest that immunization with DFTD cells polarized the immune response toward a T_H_1 or T_H_2 profile.

### Irradiated cells retain immunogenicity while sonication or freeze/thaw lysates have reduced immunogenicity

Inactivated DFTD cells that maintain their immunogenicity are required for vaccine and immunotherapy trials. To determine the best means of achieving this, BALB/c mice were injected twice IP with DFTD cells. The cells were either viable or inactivated by irradiation, sonication, or rapid freeze/thawing. Serum was collected for analysis of anti-DFTD antibodies by flow cytometry. Splenocytes from DFTD immunized mice were co-cultured with DFTD cells and the supernatant analyzed for cytokine expression.

**Figure 9:**
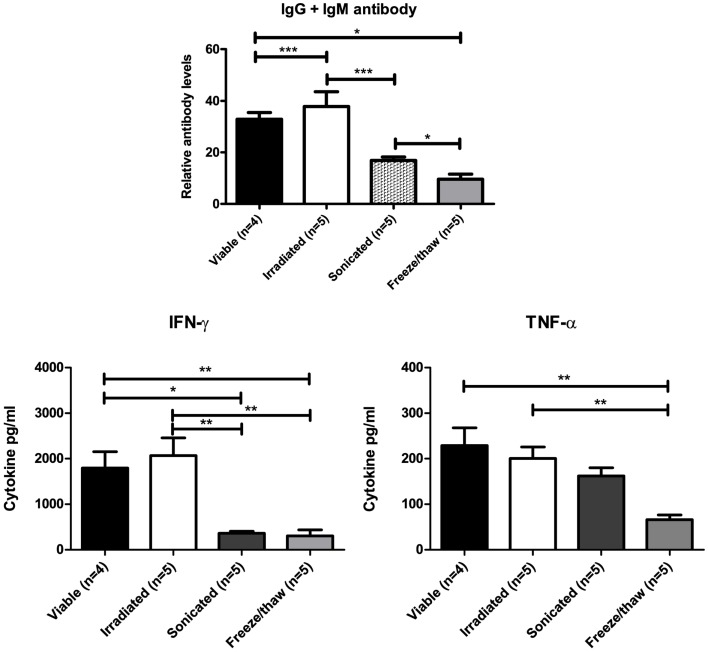
**Mice immunized with sonicated or freeze/thaw DFTD cells produced lower levels of anti-DFTD antibody and lower levels of IFN-γ and TNF-α compared to mice immunized with viable or irradiated DFTD cells** (Data are expressed as mean ± SEM, probability calculated by unpaired Student’s *t*-test, *P < 0.05, **P < 0.01, and ***P < 0.001).

Mice immunized with irradiated cells produced higher levels of DFTD specific antibodies compared to sonicated and freeze/thaw lysates. When cultured *in vitro* with DFTD cells, splenocytes from mice immunized with sonicated or freeze/thaw lysates produced lower levels of IFN-γ and TNF-α compared to splenocytes from mice immunized with irradiated cells (Figure 9). Irradiation of cells is the better method for inactivation because immunogenicity is maintained.

## Discussion

Devil Facial Tumour Disease is a cancer that is transmitted from host to host with 100% mortality and no known immunity. Lack of surface MHC-I expression on the DFTD cancer cells (8) explains the lack of allo-recognition. An inability to trigger an immune response suggests that DFTD cells are non-immunogenic and could be imperceptible to any immune system. This study focused on the fundamental question of whether DFTD cells are immunogenic and therefore it should be possible to generate an immune response against DFTD tumor cells. As access to Tasmanian devils is limited due to their endangered status and there are limited reagents such as monoclonal antibodies, we conducted this study in our mouse model of DFTD (12).

The ability to establish DFTD xenografts in immunocompromised NOD/SCID (12) mice but not in immunocompetent BALB/c and C57BL/6 mice suggests that the DFTD cells are immunogenic. To confirm that the failure to establish DFTD xenografts in immunocompetent mice was a specific immune system response, we evaluated antibody, cytokine, and cytotoxic responses in BALB/c and C57BL/6 challenged with viable DFTD cells.

Flow cytometric detection of DFTD cell surface specific antibodies in the serum of BALB/c and C57BL/6 mice provided the most robust method for detection of an immune response following immunization with DFTD tumor cells. There was no evidence for substantial levels of preformed antibodies as serum from naïve mice showed minimal binding to DFTD cells. This low level of binding could have been due to low concentrations of preformed antibodies, or simply non-specific binding. This suggests that hyperacute xenograft rejection, which depends on preformed antibodies, was unlikely to be the major cause preventing DFTD establishment.

The SC injection route for immunizations had originally been selected because of its similarity to the transfer of DFTD cells in the Tasmanian devil population. By comparing single versus multiple site SC injections, it was established that multiple site injections produced a more intense antibody response. This was most likely due to the targeting of an increased number of draining lymph nodes. Although implantations at a single site may occur frequently in wild Tasmanian devils, multiple site injections would be more effective in a vaccination program. IP injections produced an even greater and more consistent antibody response. The enhanced immunological response via the IP route in the mouse model may have implications for the induction of a protective immune response in Tasmanian devils. IP injections of vaccines for Tasmanian devils may prove more effective than SC injections. Additionally, when DFTD cells need to be inactivated for vaccine trials irradiation is preferable to sonication or freeze/thawing because the irradiated cells retain their immunogenicity in the mouse model and it is reasonable to presume this would translate to the Tasmanian devil’s responses.

Intraperitoneal injections generated both IgM and IgG responses to the DFTD cells. The switch from IgM to IgG was detected 4 and 7 days after the first immunization with DFTD cells. This is consistent with a T cell dependent antibody response to any cellular antigen. Secondary responses after a second DFTD immunization following a 95-day rest revealed an amplified IgG response. Consequently antibody responses to DFTD cells are characteristic of a typical T cell dependent humoral immune response to a cellular antigen.

The cell mediated arm of the immune response was evaluated with an *in vitro* cytotoxicity assay. Splenocytes from DFTD immunized mice produced the same level of cytotoxicity as splenocytes from naïve mice. This suggests that the response did not benefit from priming of cytotoxic T lymphocytes of the specific immune system. Cytotoxicity may be mediated by NK, NKT cells, or unprimed T cells responding to xenogeneic determinants. The significance of this finding is twofold. Cytotoxic cells can recognize DFTD cells and they can be killed. The relevance of this latter point is that as DFTD cells are susceptible to killing by murine cells they should also be susceptible to killing by cytotoxic cells of the Tasmanian devil.

Devil Facial Tumour Disease cells do not express surface MHC-I molecules (8) and therefore should be targets for NK cells. If NK cells from the mouse are the effector cells, this suggests that DFTD cells express the obligatory activating receptors. We have evidence that Tasmanian devils have NK cells (13). If mouse NK-like cells can bind to and destroy the DFTD cell *in vivo* then NK cells of the Tasmanian devil should also have the capacity to kill DFTD. The mechanism by which the DFTD cells inhibit or circumvent the NK cells of Tasmanian devils following allograft transmission of DFTD remains unknown.

With regards to NKT cells, they do not require MHC-I molecules for binding but are activated by glycolipid antigens binding to CD1d (14, 15). We have previously stated that β_2_-microglobulin is down-regulated, which is an obligatory molecule associated CD1d molecules making NKT cells unlikely effector cells. However, as β_2_-microglobulin expression can be restored in the presence of cytokines (8), NKT cells could have contributed to the immune response. It is not currently known if DFTD cells express glycolipid antigens capable of activating NKT cells.

The isotype switching of B cell antibody production is T cell dependent and directed by T cell derived cytokines resulting in antibody isotypes characteristic of either a T_H_1 or T_H_2 profile (16–18). Although an immune response against DFTD cells was demonstrated, we considered the possibility that DFTD could be a tumor cell line that biases the immune response to a T_H_2 response. This would permit tumor surveillance escape by suppressing T_H_1 anti-tumor responses (9, 19). This was analyzed by evaluating cytokines. IFN-γ, characteristic of a T_H_1 response (20), was up-regulated within days of immunization with DFTD cells. This was followed by a strong and persistent up-regulation of IL-10, which is a T_H_2 type cytokine that counterbalances the T_H_1 responses generated by IFN-γ (20). IL-10 also promotes the activation and proliferation of antigen-specific B cells (9). The timing of IL-10 up-regulation is also consistent with the detection of IgG antibodies against DFTD cells in the mice. As IgG isotypes discriminate between T_H_1 and T_H_2 responses (20), these were evaluated in the BALB/c and C57BL/6 mice. Inoculation with DFTD cells did not skew the immune response of either strain toward a T_H_1 or a T_H_2 response. There was some skewing toward IgG2a in the BALB/c mice and toward IgG2b in the C57BL/6 mice. Both strains of mice expressed high levels of IgG1, which is regarded as a T_H_2 response. The relevance of these findings is that the DFTD cells do not consistently bias the immune response toward a non-protective T_H_2 response.

As with results derived from any animal or *in vitro* model, there will be limitations and the immunological mechanisms observed may not translate to the natural host species. The combined cytokine and antibody profile in these mice demonstrates a humoral response to DFTD, which is consistent with xenograft rejection mechanisms involving NK cells (21). Xenograft rejections are MHC-I independent and allograft rejections are based upon MHC-I incompatibilities. DFTD cells do not express surface MHC-I (8) and therefore cannot be rejected by MHC incompatibilities. Rejection must therefore rely on other cell types such as NK cells or NKT cells. NK cells are the most likely cell type involved in the rejection of the DFTD xenograft described in this paper. Clearly, Tasmanian devil NK or NKT cells are not targeting the DFTD tumor cells. But as mouse cells can target DFTD, immunogenic membrane signals would be present to act as targets.

In conclusion, the main finding of this study is that DFTD cells are suitable targets for immunotherapy. There is a need to develop a vaccine or treatment to prevent extinction of the Tasmanian devil in the wild and various strategies can be trialed in the mice before moving to clinical trials with Tasmanian devils.

## Conflict of Interest Statement

The authors declare that the research was conducted in the absence of any commercial or financial relationships that could be construed as a potential conflict of interest.
